# Upper cervical two-point discrimination thresholds in migraine patients and headache-free controls

**DOI:** 10.1186/s10194-018-0873-z

**Published:** 2018-06-26

**Authors:** Kerstin Luedtke, Waclaw Adamczyk, Katrin Mehrtens, Inken Moeller, Louisa Rosenbaum, Axel Schaefer, Janine Schroeder, Tibor Szikszay, Christian Zimmer, Bettina Wollesen

**Affiliations:** 1 Department of Human Movement Science, University of Hamburg, Hamburg, Germany; 2 Academic Physiotherapy, Medical Section, Department of Orthopaedics and Trauma Surgery, University of Luebeck, Luebeck, Germany; 3 The Jerzy Kukuczka Academy of Physical Education, Department of Physiotherapy, Katowice, Poland; 4 Faculty of Social Science, Degree Course Speech and Language Therapy and Physiotherapy, University of Applied Sciences Bremen, Bremen, Germany; 5 Pain Research Group, Institute of Psychology, Jagiellonian University, Krakow, Poland; 6 Department of Systems Neuroscience, University Medical Center Hamburg-Eppendorf, Hamburg, Germany

**Keywords:** Tactile acuity, Hypersensitivity, Migraine, Headache, Two-point discrimination

## Abstract

**Background:**

Chronic pain including migraine is associated with structural and functional changes in the somatosensory cortex. Previous reports proposed two-point discrimination (TPD) as a measurement for cortical alterations. Limited evidence exists for tactile acuity in the neck and no data is available for migraine.

**Methods:**

To introduce a standardized protocol for the measurement of TPD in the upper cervical spine, 51 healthy participants were investigated with a newly developed paradigm which was evaluated for intra-rater reliability. The same protocol was applied by two further examiners to 28 migraine patients and 21 age-, and gender-matched healthy controls to investigate inter-rater reliability and between group differences.

**Results:**

Results indicated excellent intra-rater (right ICC_(2,4)_ = 0.82, left ICC_(2,4)_ = 0.83) and good inter-rater reliability (right ICC_(2,4)_ = 0.70, left ICC_(2,4)_ = 0.75). Migraine patients had larger TPD thresholds (26.86 ± 7.21) than healthy controls (23.30 ± 6.17) but these became only statistically significant for the right side of the neck (*p* = 0.02). There was a significant, moderate association with age for the right side (*r* = 0.42 *p* = 0.002, *n* = 51), and less strong association for the left side (*r* = 0.34, *p* = 0.14) in healthy individuals. TPD did not correlate with headache days per month or the dominant headache side in migraine patients.

**Conclusions:**

Surprisingly, migraine patients showed increased TPD thresholds in the upper cervical spine interictally. Although a body of evidence supports that hypersensitivity is part of the migraine attack, the current report indicates that interictally, migraine patients showed worse tactile acuity similar to other chronic pain populations. This has been hypothesized to indicate structural and functional re-organisation of the somatosensory cortex.

## Background

Current knowledge on functional and structural cortical alterations following longstanding pain, is mainly based on chronic low back pain populations [1–6] with very limited data for the upper spinal region [7]. However, it has been suggested, that at least structural brain changes are similar for any chronic pain condition including headaches [8] and that the somatosensory cortex is one of the structures typically affected [5, 9, 10].

Two-point discrimination threshold (TPD) assessment is a routine procedure during the neurological examination that has been shown to be associated with such structural brain changes [9, 10]. TPD thresholds indicate the smallest distance between two points of sliding mechanical callipers, that can still be perceived as two distinct points. This paradigm has been used in research to investigate tactile acuity in chronic pain patients and a recent systematic review reported a general tendency to increased TPD thresholds in patients with longstanding pain compared to pain-free controls [11, 12].

Six studies have previously investigated TPD in the neck: Song et al. investigated patients with spinal cord injuries and found multiple sites of reduced sensory discrimination compared to healthy controls that were reported to be particularly distinct in the neck area [13]. Moreira et al. found increased TPD thresholds in patients with idiopathic neck pain but although there was a mean difference between groups of almost 5 mm, values failed to reach statistical significance [14]. Nolan et al. investigated TPD only in healthy participants; the mean TPD at the level of C7 was 55.4 mm [15]. All three studies applied their measurements to the lower cervical area. The only study that included a measurement in the upper cervical spine in patients with mild recurrent neck pain, while observing slightly larger TPD values in the patient group, failed to show any statistically significant difference between patients and controls at C2 and at C7 [16]. The mean two-point discrimination threshold of the included 30 neck pain patients was 29.75 mm at C2 and 32.5 mm at C7. Catley et al. and Harvie et al. conducted reliability studies in healthy participants. The authors examined TPD threshold only at the C7 spinal level and reported a mean of 45.9 mm and of 35 mm, respectively [17, 18].

All six studies showed that tactile acuity can feasibly be measured in the cervical area, two studies confirmed the intra-rater and inter-rater reliability (ICC > 0.75) of the measurement [17, 18]. All studies evaluating patients showed that the patient population had larger TPD values compared to the control group. No study has previously applied TPD to a headache population. However, the test procedures used in the six studies were heterogeneous and reported a wide range of mean threshold in a variety of populations. Only one study investigated the upper cervical region and no data were available for migraine patients.

The aim of the current project was therefore to investigate TPD thresholds in the upper cervical spine in patients with migraine in comparison to healthy controls. Since only one study has investigated this region previously, this current project includes the development and both, intra- and inter-reliability testing of the procedure.

## Methods

### Study overview

**Fig. 1:**
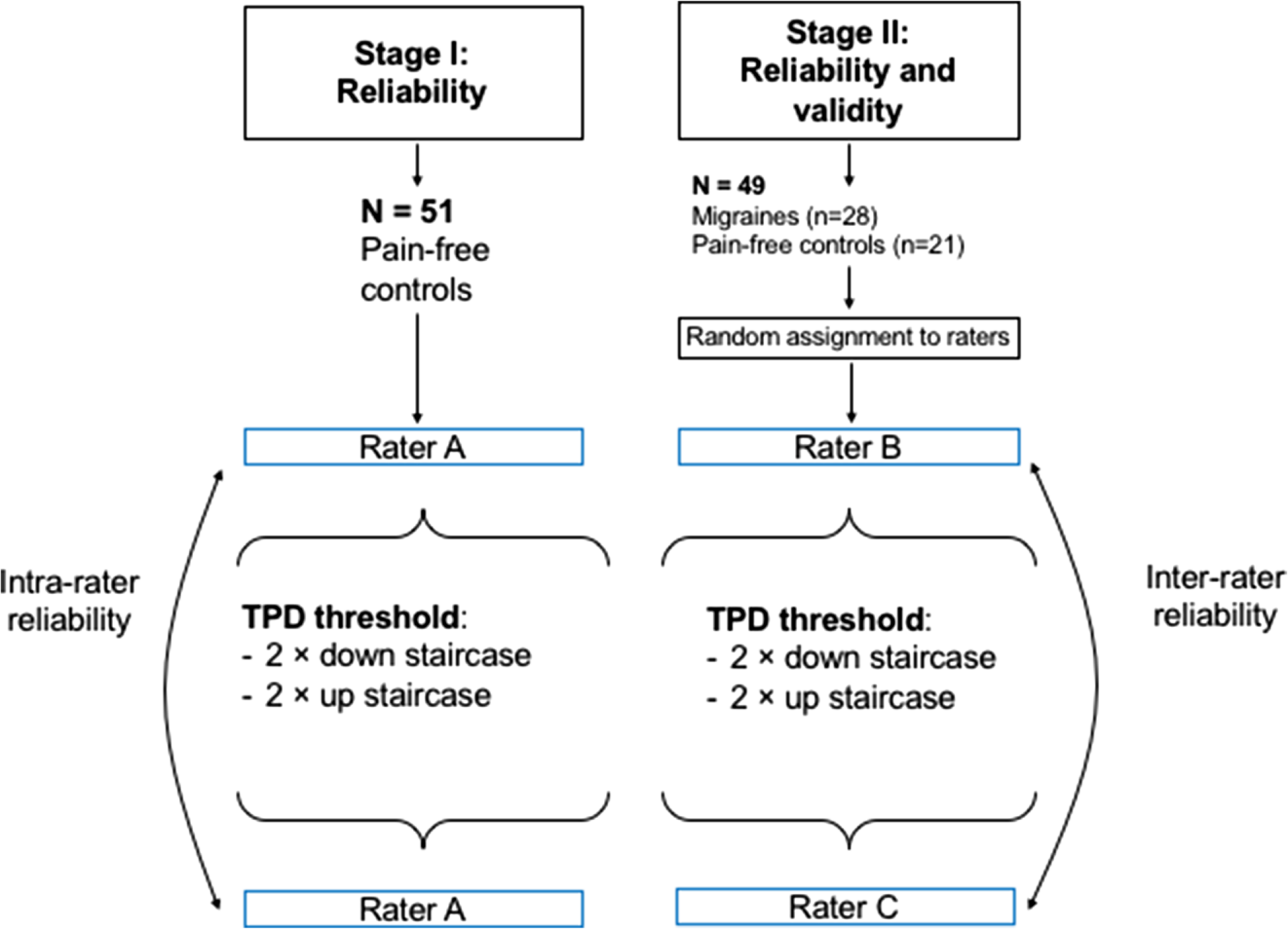
Study design. Intra-rater reliability was assessed in the first stage (left) while inter-rater and differences between migraines and controls in the second stage of the study. TPD - Two-point discrimination threshold

The study was designed in two stages: the initial stage was the development of the test protocol and determination of intra-rater reliability using a test-retest design. The second stage for the inter-rater reliability and the difference between migraine patients and controls was a repeated-measures case-control design with blinded examiners (Fig. 1).

The study was approved by the local ethics committee of the University of Hamburg (AZ 2017_86). It was registered a priori in the German clinical trials register (DRKS00011795). The data collection for the intra-rater stage was conducted between November and December 2016 and for the inter-rater and case control stage between May and June 2017.

### Participants

Healthy control participants for the intra-rater reliability phase as well as those serving as controls for the case-control study had to be adults (18+ years old), age and gender matched to the patient group (only for the case-control phase), and right-handed. Patients for the case-control phase were: adults, diagnosed with migraine according to IHS 3 criteria [19], had a minimum of 4 migraine days on average per month, took pain medication on < 10 days per month to exclude medication overuse headache, had no other relevant headache diagnosis (episodic tension-type headache was accepted), had to be headache-free 48 h prior to the assessment. Patients and controls were excluded when they were older than 65 years; to reduce bias and to differentiate between the influence of headache and any other pain condition, they were also excluded when they had acute pain including toothache on the day of testing, had any chronic pain condition, had neck pain in the past 3 months, had a history of cervical pathology or trauma including whiplash associated disorder, disc disease or others requiring medical intervention, suffered from any psychological, neurological or other disease potentially influencing the sensory system, or a skin condition in the area over the upper cervical spine.

Patients were recruited from the University Headache Clinic at the University Medical Center Hamburg-Eppendorf and from local neurology clinics. Control participants were selected from social media and digital network postings and personal contacts.

### Measurement procedure

An initial test protocol was developed and piloted in a small convenience sample of healthy participants before it was subsequently applied to the study population. It was based on the most widely used procedure for the lumbar spine of Moberg [20] who introduced a protocol with increasing and decreasing distances between two tips of a mechanical sliding calliper [20] similar to the *method of limits* used in the quantitative sensory testing protocol [21]. Previous publications suggested that the discrimination threshold in the neck will be between 10 and 45 mm [11, 15, 18] and it was reported as 29.75 mm at C2 in patients with neck pain [16]. Therefore, the TPD test started at a distance of 50 mm, a distance that was perceived by all participants as two distinct points. The distance was subsequently decreased by 5 mm until only 1 point was perceived and increased in steps of 1 mm until two points were felt. This was followed by decreasing the distance in 1 mm steps until only one point was perceived. This was repeated until 4 values were documented, two from increasing and two from decreasing the distance. The mean of these 4 data points was the TPD threshold for one measurement. For the intra-rater evaluation, the procedure was repeated by the same examiner after a five-minute break. All participants were tested on both sides of the neck in a randomized order.

Participants were positioned on a stool in front of a table with the forehead resting on a folded towel. The third vertebra was located manually and marked with a felt tip pen. The medial tip of the callipers was always within a radius of 2 cm from the midpoint of the 3rd vertebrae. The TPD was always measured horizontally at the level of C3. To standardize the pressure, calliper tips were placed on the test location until the first blanching of the skin. This standardization has been widely applied in previous studies assessing tactile acuity [17, 22].

For the inter-rater reliability and case-control stage, patients and controls were randomly allocated to a first blinded examiner who performed the TPD testing as described above (two measurements on each side of the neck, 1 measurement at the hand). After a resting period of 10 min they changed to the second blinded examiner. During this stage of the study, the lateral border of the dorsal hand was additionally tested as a control region in both groups, healthy controls and migraines. This allowed an indication whether tactile acuity changes are limited to the painful region or can also be identified in remote regions, thereby potentially indicating central sensitization.

Blinding of the examiners was ensured by a third researcher, who contacted potential participants, performed the randomization to the first blinded examiner, distributed the questionnaires and instructed participants to not reveal their status as a patient or a control person during the test procedure. Both examiners were experienced in the physical examination of patients and trained during repeated sessions to perform the test in a standardized manner. In addition to the TPD thresholds, the following parameters were recorded during the test days by a piloted questionnaire: gender, age, number of headache days per month, years since diagnosis and dominant headache side.

### Proposed sample size and statistical analysis

The required sample size for the case-control phase was based on data for patients with chronic low back pain [13, 22] pain using 80% power and an alpha value of 0.05. The minimal required sample size per group was minimum of 19, the calculation was conducted in G*Power [23].

Reliability was calculated using the two-way random average measures intraclass-correlation coefficient for absolute agreement (ICC_2,4_). Reliability was interpreted as excellent if the ICC was > 0.75 [24] and good if the ICC was between 0.74–0.50. The variance components (σ^2^ patient, σ^2^ observer and σ^2^ residual) were calculated with VARCOMP-Analysis (Method: ANOVA Type III Sum of Squares) to determine Standard Error of Measurement (SEM) and the smallest detectable difference (SDD) [25]. Differences across groups were calculated using two-sided t-tests for independent samples for each side of the neck and for the reference area (hand) individually. For these calculations, the mean ratings across the two examiners were used.

Since previous research has indicated that age and gender may play a role in the TPD measurement [26, 27], secondary analyses evaluated whether TPD thresholds were significantly different between male and female patients and in younger and older patients. For the analysis of age, a median split was used and significance was tested using t-tests at an alpha level of 0.05. Additionally, Pearson’s correlation coefficient was calculated to assess association between age and TPD threshold. Because TPD in the neck region has recently been shown to be associated with the severity of a disease [28], TPD thresholds were correlated in the headache group with headache days per months and with the dominant headache side using Pearson’s correlation. All analyses were calculated using the software packages SPSS 23 (IBM, Illinois, USA) and Stata 15 (StataCorp LLC, College Station, Texas, USA).

## Results

### Intra-rater reliability

Fifty-one healthy participants (26 female) aged between 20 and 65 years (mean 37.8 ± 13 years) were recruited. TPD thresholds ranged between 7.5 and 41 mm (mean 26.7 ± 7.1 mm). Values for male and female as well as older and younger participants are presented in Table 1. The intra-rater reliability was excellent with an ICC_(2,4)_ of 0.83 (95% CI, 0.72–0.90) for the left side of the neck and an ICC_(2,4)_ of 0.82 (95% CI, 0.70–0.89) for the right side of the neck. The SEM was 3.1 mm for both sides with corresponding SDDs of 8.5 (left side) and 8.6 (right side). There was a significant, moderate correlation between age and TPD (left side *r* = 0.342; right side *r* = 0.421).

**Table 1:** TPD thresholds for male and female as well as younger and older participants (intra-rater reliability sample)

Between group differences	Male (*n* = 25)	Female (*n* = 26)	*t* - test
Left neck (mean ± SD)	2.75 (0.71) cm	2.6 (0.72) cm	*p* = 0.458
Right neck (mean ± SD)	2.9 (0.73) cm	2.5 (0.53) cm	*p* = 0.031
Between group differences	Age 36 ≥ (n = 26)	Age > 35 (n = 25)	*t* - test
Left neck (mean ± SD)	2.42 (0.64) cm	2.93 (0.7) cm	*p* = 0.008
Right neck (mean ± SD)	2.40 (0.54) cm	3.0 (0.65) cm	*p* = 0.001

*SD* Standard deviations

### Inter-rater reliability and case-control

For the inter-rater and case-control phase of the study, 49 participants (28 migraine patients) were recruited. Characteristics for patients and control participants are reported in Table 2. The inter-rater reliability for all measurements on the left (ICC_(2,4)_ 0.75; 95% CI 0.55–0.86; SDD 12.02; SEM 4.34) and the right side (ICC_(2,4)_ 0.70; 95% CI 0.46–0.83; SDD 12.36; SEM 4.46) of the neck and for the measurements on the hand (ICC_(2,4)_ 0.70; 95% CI 0.48–0.83; SDD 6.01; SEM 2.1) was good. Migraine patients had larger TPD thresholds at all test locations including the hand. Statistical significance was only reached for the right side of the neck (*p* = 0.02) (Table 3). There was no correlation with headache days per month for left (*r* = − 0.09), or right side (*r* = − 0.11) of the neck, and the hand (*r* = − 0.15). Neither was a correlation with the dominant headache side revealed for the left (r = − 0,19) or the right side (r = − 0,20) of the neck.

**Table 2:** Characteristics of groups

Variable	Migraine *n* = 28	Control participants *n* = 21
Female: number (%)	27 (93.1%)	17 (85%)
Male: number (%)	2 (6.89%)	3 (15%)
Age (years)	34.4 (11.9)	39.8 (13.6)
Years since diagnosis	19.7 (14.1)	–
Headache days per month	9.4 (9.1)	–
Dominant headache side		
- Left number (%)	7 (24.1)	–
- Right number (%)	7 (24.1)	–
- Bilateral number (%)	15 (51.7)	–

**Table 3:** Mean and standard deviations of two-point discrimination thresholds

Side / location of testing	Migraine patients (*n* = 28)	Healthy control (*n* = 21)	*t* - test
Left	27.4 (8.2) mm	24.9 (7.1) mm	*p* = 0.27
Right	26.3 (7.3) mm	21.7 (6.2) mm	*p* = 0.02
Hand	8.3 (4.0) mm	6.4 (2.2) mm	*p* = 0.05

## Discussion

This paper aimed to investigate tactile acuity in migraine patients compared to headache-free controls and to develop a standardised test protocol with sufficient intra- and inter-rater reliability. The mean TPD thresholds in healthy participants in this study are in line with those in the only other study on the upper cervical spine [16]. Intra- and inter-rater reliability coefficients were similarly high as those reported by Harvie et al. [28] and Catley et al. [17] for the lower part of the neck, thereby supporting TPD testing as a clinically reliable tool. Also similar to the results reported by Harvie et al. [28], who assessed patients with chronic neck pain, migraine patients scored worse, i.e. had larger TPD thresholds than control participants, although the results in this current study were only statistically significant for the right side of the neck. TPD thresholds for the left side of the neck were also larger than in healthy controls but not statistically significant. Furthermore, results did not overcome the SDD and were very close to the SEM and might therefore be in line with Elsig et al. who found no difference between patients with recurrent neck pain and healthy controls [16]. The consistency of the direction of change for all tested body regions and the overwhelming majority of publications in this field showing larger TPD thresholds in pain patients compared to healthy populations, increase the confidence in the current results. Interestingly, although not statistically significant was, that TPD thresholds were also increased when measured outside the area of clinical pain at the right hand, pointing towards central rather than peripheral or region-specific changes. This result is also similar to that reported by Harvie et al. [28]. Older and younger healthy participants (median split at 35 years) showed a statistically significant difference and younger females showed the highest tactile acuity, i.e. smallest TPD thresholds.

Although significance was only reached for the right side of the neck, the generally increased TPD thresholds in migraine patients compared to controls might support the frequently stated hypothesis and that repeated migraine attacks result in similar cortical changes as observed in patients with chronic pain conditions [11]. While not surprising in the context of chronic pain research, the results of the current study are somewhat puzzling considering the prevalence of hypersensitivity and/or allodynia in patients with migraine. Altered sensitivity to external stimuli such as light, noise, smell or touch is one of the trait symptoms and diagnostic criteria for migraine [19]. Numerous studies focused on this phenomenon and reported hypersensitivity and/or allodynia in patients with migraine [29–37]. Smaller TPD thresholds would have been the more intuitive result representing an increased tactile sense and thereby a phenomenon which could be interpreted as hypersensitivity. One explanation for this controversy could be that hypersensitivity and/or allodynia are indeed only present during the pre-ictal and ictal phase of migraine [33], while reports on interictal hypersensitivity are conflicting [38]. A further explanation could be that allodynia and/or hypersensitivity is restricted to the reference area of the trigeminal nerve. Very few studies, additionally to the typically measured supraorbital region, measured hypersensitivity in the neck [34, 39, 40]. This limited evidence is surprising, since neck pain is more common in migraine patients than other typically associated symptoms - such as nausea - and occurs in more than 70% of migraine patients [41]. The 12-item Allodynia Checklist contains items such as “wearing a neckless” which was found to be the second most discriminative item between migraine patients with and without cutaneous allodynia [36]. Based on the trigeminocervical convergence theory, stating that afferent fibres from the trigeminal and the cervical systems connect to the same nuclei in the brainstem, there is a strong anatomical and physiological explanation for a reciprocal influence of the trigeminal system on cervical reference areas and vice versa [42–44]. Furthermore, a study using a neural staining technique in rodents reported a connection between the dura mater and peripheral muscles through the cranial fissures and thereby another possible pathway explaining neck pain or/and sensitivity when the dura mater is sensitized [45].

A final explanation is that tactile acuity and hypersensitivity/allodynia, while fulfilling the same biological role of an increased awareness to potentially damaging stimuli to a diseased or painful body region, are unrelated entities. The most common paradigm to quantify hypersensitivity and/or allodynia is the quantitative sensory testing (QST) protocol where different noxious and non-noxious modalities are applied to the affected body part and perception thresholds, pain thresholds and pain responses are recorded [21]. However, QST requires equipment not always available to clinicians and the full protocol is extremely time consuming. Whether TPD thresholds can be used as an alternative test paradigm feasible for the use in the daily clinical practice remains to be evaluated in future studies.

A limitation of this study is, that only the neck and the hand but not the reference area of the trigeminal nerve was assessed. Although this was the aim of this project, retrospectively, an additional assessment of the supraorbital area on the side most affected by migraine would have been helpful to at least partially disentangle whether the test location influences results. Furthermore, a second modality, such as thermal thresholds or von Frey hairs would have been interesting to compare the current results with previous publications and to investigate whether TPD thresholds are related to hypersensitivity and/or allodynia. To disentangle responses to tactile stimuli and thereby better understand what the test modalities actually measure would be valuable future projects.

## Conclusion

Despite the limited available space in the upper cervical region, TPD thresholds can be measured reliably in healthy participants and in patients with migraine. Younger and female patients have smaller TPD thresholds than older and male patients, future research should take this into account. Patients with migraine show larger TPD thresholds in the upper cervical region, but significance was only reached for the right side of the neck. Remote body parts also showed increased values (but did not reach statistical significance), potentially pointing towards cortical changes similar to those reported for chronic pain conditions.

## Abbreviations

ICC: Intraclass correlation coefficient
QTS: Quantitative sensory testing
SD: Standard deviation
SEM: Standard error of measurement
TPD: Two-point discrimination threshold
